# High prevalence and diversity of HIV-1 non-B genetic forms due to immigration in southern Spain: A phylogeographic approach

**DOI:** 10.1371/journal.pone.0186928

**Published:** 2017-10-30

**Authors:** Santiago Pérez-Parra, Natalia Chueca, Marta Álvarez, Juan Pasquau, Mohamed Omar, Antonio Collado, David Vinuesa, Ana Belen Lozano, Gonzalo Yebra, Federico García

**Affiliations:** 1 Servicio de Microbiología Clínica, Hospital Universitario San Cecilio, Campus de la Salud e Instituto de Investigación IBS, Granada, Spain; 2 Servicio de Infecciosas, Hospital Virgen de las Nieves, Granada, Spain; 3 Servicio de Infecciosas, Hospital Ciudad de Jaén, Jaén, Spain; 4 Servicio de Medicina Interna, Hospital de Torrecárdenas, Almería, Spain; 5 Servicio de Infecciosas, Hospital Universitario San Cecilio, Granada, Spain; 6 Servicio de Infecciosas, Hospital de Poniente, Almería, Spain; 7 The Roslin Institute, University of Edinburgh, Edinburgh, the United Kingdom; University of Cincinnati College of Medicine, UNITED STATES

## Abstract

Phylogenetic studies are a valuable tool to understand viral transmission patterns and the role of immigration in HIV-1 spread. We analyzed the spatio-temporal relationship of different HIV-1 non-B subtype variants over time using phylogenetic analysis techniques. We collected 693 *pol* (PR+RT) sequences that were sampled from 2005 to 2012 from naïve patients in different hospitals in southern Spain. We used REGA v3.0 to classify them into subtypes and recombinant forms, which were confirmed by phylogenetic analysis through maximum likelihood (ML) using RAxML. For the main HIV-1 non-B variants, publicly available, genetically similar sequences were sought using HIV-BLAST. The presence of HIV-1 lineages circulating in our study population was established using ML and Bayesian inference (BEAST v1.7.5) and transmission networks were identified. We detected 165 (23.4%) patients infected with HIV-1 non-B variants: 104 (63%) with recombinant viruses in *pol*: CRF02_AG (71, 43%), CRF14_BG (8, 4.8%), CRF06_cpx (5, 3%) and nine other recombinant forms (11, 6.7%) and unique recombinants (9, 5.5%). The rest (61, 37%) were infected with non-recombinant subtypes: A1 (30, 18.2%), C (7, [4.2%]), D (3, [1.8%]), F1 (9, 5.5%) and G (12, 7.3%). Most patients infected with HIV-1 non-B variants were men (63%, p < 0.001) aged over 35 (73.5%, p < 0.001), heterosexuals (92.2%, p < 0.001), from Africa (59.5%, p < 0.001) and living in the El Ejido area (62.4%, p<0.001). We found lineages of epidemiological relevance (mainly within Subtype A1), imported primarily through female sex workers from East Europe. We detected 11 transmission clusters of HIV-1 non-B Subtypes, which included patients born in Spain in half of them. We present the phylogenetic profiles of the HIV-1 non-B variants detected in southern Spain, and explore their putative geographical origins. Our data reveals a high HIV-1 genetic diversity likely due to the import of viral lineages that circulate in other countries. The highly immigrated El Ejido area acts as a gateway through which different subtypes are introduced into other regions, hence the importance of setting up epidemiological control measures to prevent future outbreaks.

## Introduction

The high evolutionary rate and recombination capacity of human immunodeficiency virus type 1 (HIV-1) determine the existence of an array of subtypes and recombinant forms circulating worldwide [1–4]. HIV-1 non-subtype B (“non-B”) variants cause around 90% of infections worldwide, and largely predominate in African or Eastern European countries with generalized HIV-1 epidemics. Subtypes C and A, and circulating recombinant forms (CRF) CRF01_AE and CRF02_AG, are responsible alone for 70% of the world’s infections [5]. Nowadays the proportion of infections by HIV-1 non-B variants in Spain lies at 12–15%, depending on the study and technique used to characterize variants [6,7]. Nonetheless, the predominance of HIV-1 subtype B in developed countries (where antiretroviral therapy is more widespread), implies that this is the most widely studied subtype from the genetic, biological and therapeutic viewpoints. The full biological meaning of the genetic variability of HIV-1 is still not completely understood. However, several major differences between the biological properties of certain genetic subtypes in have been described; e.g., virulence, tropism and transmissibility [8,9], use of chemokine co-receptors [10], disease progression [11], susceptibility to some antiretroviral drugs [12,13], sensitivity to viral load quantification methods [14,15] and detection [16]. These findings evidence the importance of epidemiological information about different subtypes.

Eastern Andalusia is located in south-eastern Spain, and includes the provinces of Almería, Granada and Jaén. Given its location and geographic closeness to the African continent, this region has received a notable foreign migratory influx in the last decade. Andalusia is the fourth Spanish Autonomous Community in number of foreign population, only surpassed by Catalonia, Madrid and the Valencian Community. The main source of immigration in Eastern Andalusia stems from its intensive farming practices, mainly in the El Ejido area (located in the province of Almería), where one in every four citizens is an immigrant.

Phylogenetic analyses, in conjunction with geographical data, can assess the existing relationship between migratory events and spread of HIV-1 on a local scale [17–20], and to study HIV-1 transmission networks locally [21–24]. As in previous studies [25], our center collects the HIV-1 *pol* gene sequences linked to the patients’ clinical data to monitor baseline drug resistance in naïve individuals in Eastern Andalusia. Our aims were to describe the molecular epidemiology and evolutionary history of non-B forms in Eastern Andalusia over the 2005–2012 period, and to explore their putative geographical origin prior to their arrival to our region.

## Methods

### Study population

During the study period (2005–2012), 693 *pol* gene sequences of patients newly diagnosed with HIV-1 in different Eastern Andalusian hospitals were collected from routine drug resistance analyses. These hospitals were distributed in 3 provinces: Granada (which included its capital city of Granada and Motril), Jaén, and Almería (including its capital city of Almería and El Ejido). The *pol* sequences (protease (PR), codons 4–99; reverse transcriptase (RT), codons 38–247) obtained by the Trugene® HIV Genotyping kit (Siemens, NAD), were linked to demographic (risk group, age, gender, country of origin, sampling year, and attending hospital), clinical (CD4+ T-cell count) and virological (plasma viral load) information. Demographic information was voluntarily collected during clinical interviews. This study was approved by the San Cecilio Hospital’s Ethics Committee, and no consent information was required as patient information remained anonymous and was de-identified prior to analyses.

### HIV-1 *pol* sequencing and subtype assignment

All the sequences were trimmed to 883 nucleotides (nt) and aligned using ClustalW [26]. The viral subtype was studied with the REGA v3.0 subtyping tool (http://dbpartners.stanford.edu:8080/RegaSubtyping/stanford-hiv/typingtool/), and was confirmed by phylogenetic analysis through maximum likelihood (ML) using the randomized Accelerated Maximum Likelihood (RAxML) program, accessible on the CIPRES Science Gateway [27]. The general time-reversible (GTR) model with a gamma-distributed heterogeneity rate across sites was employed, applying 1000 bootstrap iterations. A representative dataset of HIV-1 group M sequences, including non- recombinant subtypes (A-K) and recombinant forms (at least four representative sequences of each non-recombinant subtype and the CRF currently available from the analysis) were downloaded from the Los Alamos HIV sequence database (http://www.hiv.lanl.gov) was used as a reference dataset (S1 Table).

The assignment to any subtype/CRF was considered definitive if the query sequence was included with the reference sequences corresponding to that viral variant in a monophyletic cluster supported by high bootstrap values (>70%) [28]. Any genetic form not associated with reference subtypes/CRFs was classified as a unique recombinant form (URF), whose recombination pattern was further studied by a Bootscan analysis using the SimPlot v3.5.1 software [29]. The bootscanning method in SimPlot consists of a sliding-window phylogenetic bootstrap analysis of the query sequence aligned against a set of reference strains to reveal breakpoints. The Neighbor-Joining algorithm was selected, with the Kimura 2-parameter substitution model. We employed a window size of 200nt moving in 10nt increments. We used a minimum cutoff for the bootstrap value of 70% to reliably assign each of the breakpoint segments to a parental variant.

We have submitted to GenBank the major groups of HIV-1 non-B variants under accession numbers MF628109 to MF628250. These were defined as those found in at least five patients. With the aim of protecting the identity of patients infected with rare genetic forms of HIV-1, and for similar scientific and ethical reasons as explained in other HIV cohorts [30–32], we decided not to submit to GenBank those sequences corresponding to the less frequent variants.

### Inference of the putative geographical origins of the HIV-1 non-B variants circulating in Andalusia

To further characterize the relationships among the major groups of HIV-1 non-B variants, we interrogated GenBank for genetically related sequences to our major subtypes/recombinant forms using HIV-BLAST (http://www.hiv.lanl.gov/content/sequence/BASIC_BLAST/basic_blast.html). The 10 most closely related GenBank sequences to each of our study sequences, were downloaded and included in each dataset. We also included all the *pol* sequences (start: 2293 and end: 3290, HXB2 coordinates), available in the HIV Los Alamos database sampled in Spain for each dataset: subtype A1 (n = 60), subtype C (n = 52), subtype F (n = 143), subtype G (n = 64), CRF14_BG (n = 25), and CRF02_AG (n = 265). Since very few sequences for CRF06_cpx were available in public databases (http://www.hiv.lanl.gov/content/sequence/HIV/mainpage.html), we included them all (n = 110).

All these individual sequence datasets were put together (n = 970) and a global phylogenetic analysis was performed using RAxML (GTR + Gamma model) and 1000 bootstrap iterations for this analysis. The phylogenetic relatedness between the sequences was studied, and a 70% bootstrap value was taken as a significantly reliable value [28]. Thresholds for low genetic distance, which are commonly used as a proxy for divergence time, were not applied to the cluster definition in the ML trees since these clusters were further confirmed and analyzed using a time-stamped Bayesian phylogenetic analysis with BEAST, as described below. International non-B lineages (defined as phylogenetic associations of at least one sequence from our cohort clustered with sequences from different countries), and ‘Andalusian clusters’ (monophyletic associations of sequences in our cohort alone), were identified in the global ML tree.

A Bayesian Markov Chain Monte Carlo (MCMC) approach was applied to each of the individual HIV-1 non-B subtype/CRFs datasets described above, which included the most genetically similar sequences found with HIV-1 BLAST, as implemented in BEAST v1.7.*5* [33]. The Shapiro-Rambaut-Drummond-2006 (SRD06) substitution model was used, together with a relaxed uncorrelated lognormal clock (UCLN)[34] and a demographic non parametric model, Bayesian Skyline Plot (BSP) [35]. This model combination was chosen because it best fits the analysis of the HIV-1 *pol* data run in the majority of studies [36]. The MCMC was run for 250 million states sampling every 50000. The evolutionary rate (μ, nucleotide substitutions per site per year, subst./site/year) for the different HIV-1 non-B subtypes/CRFs (S2 Table), and the most recent common ancestors (MRCA) of the different HIV-1 non-B clusters, were estimated. Only traces with an effective sample size (ESS) > 200 for all the parameters, after excluding an initial 10% burnin, were accepted as visualised in TRACER, v1.6 (http://tree.bio.ed.ac.uk/software/tracer/).

Maximum Clades Credibility (MCC) trees were constructed in each case to summarise the posterior tree distributions. In these MCC trees, the more epidemiologically relevant clusters and lineages, previously identified in the global ML tree, were studied; and a node support cutoff (posterior probability (pp) above 0.9) was applied for their confirmation. Trees were viewed and edited in FigTree, v. 1.4.0 (http://tree.bio.ed.ac.uk/software/figtree).

### Analysis of the antiretroviral drug resistance mutations

Drug resistance mutations were identified in the *pol* sequences using the HIVseq program, which is available in the HIV Drug Resistance Database of Stanford University (https://hivdb.stanford.edu/hivseq/by-sequences/), and also using the WHO surveillance drug resistance mutation list (last updated in 2009 by Bennett and colleagues) [37].

### Statistical analyses

A multivariate logistic regression analysis was performed to determine the predictive effect of the demographic, clinical and virological characteristics on the adscription to each subtype/CRF. The statistical significance of these characteristics, compared to the total proportion of infected patients, was studied by a hypothesis contrast using a z-test. The statistical analysis was performed with SPSS 22.0.

## Results

### Epidemiological surveillance of the non-B HIV-1 genetics forms

**Fig 1:**
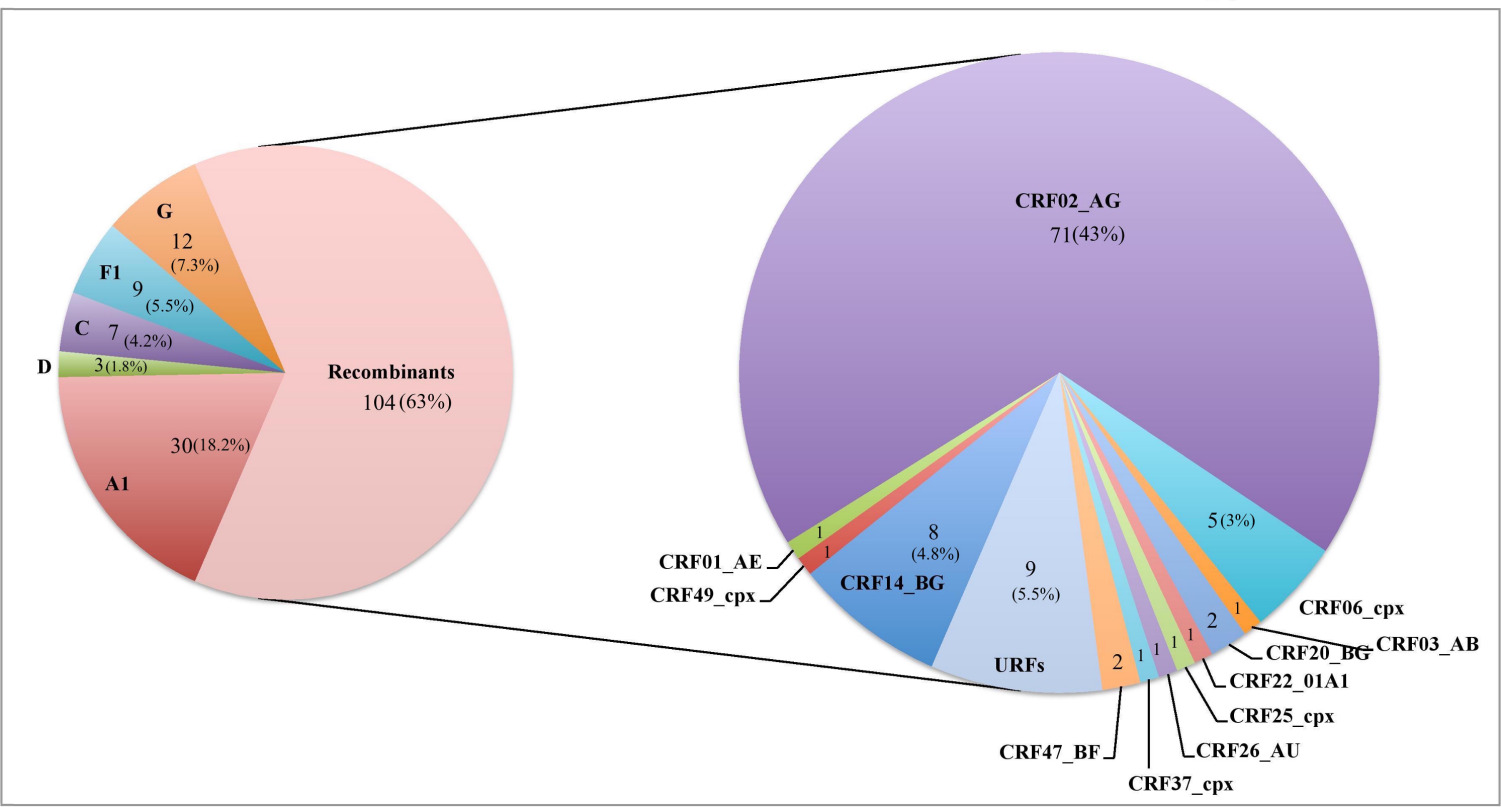
Distribution of the HIV-1 non-subtype B genetic forms detected in Eastern Andalusia over the 2005–2012 period.

Of the 693 total included patients, 165 (23.8%) were infected with different genetic forms of HIV-1 non-B variants. Most of them (n = 104, 63%) were recombinant viruses in *pol*: 95 (57.6%) corresponded to 12 different CRFs and nine (5.5%) were URFs. The other patients (n = 61, 37%) were infected with five non-recombinant subtypes: A1, D, C, F1 and G (see Fig 1).

The demographic, clinical and virological characteristics of the patients according to the genetic HIV-1 non-B forms are provided in Table 1. Most of the patients infected with non-B variants were men (63%, p < 0.001) aged over 35 (73.5%, p < 0.001), heterosexual (92.2%, p < 0.001), African (58.2%, p < 0.001), and living in the El Ejido area (62.4%, p<0.001). The full list of countries of origin for patients infected with non-B forms and born abroad (n = 127 [77%]) were: Argentina, n = 1, Brazil, n = 5, Burkina Faso, n = 1, Cameroon, n = 1, Colombia, n = 1, Congo, n = 5, Ivory Coast, n = 1, Cuba, n = 1, Gambia, n = 2, Ghana, n = 13, Guinea, n = 10, Guinea-Bissau, n = 11, Equatorial Guinea, n = 4, Lithuania, n = 1, Mali, n = 9, Morocco, n = 3, Mauritania, n = 1, Nigeria, n = 17, Dominican Republic, n = 1, Romania, n = 6, Russia, n = 14, Senegal, n = 15, Sierra Leone, n = 1 and South Africa, n = 3. The rest of subjects (n = 36, 22%) had been born in Spain.

**Table 1:** Demographic, clinical and virological characteristics of the patients infected with HIV-1 non B variants sampled over the 2005–2012 period.

	NON-RECOMBINANT SUBTYPES				RECOMBINANT FORMS				
CHARACTERISTICS	A1	C	F1	G	CRF02_AG	CRF14_BG	CRF06_cpx	Others	Total
**Gender**									
Male	10 (33.3)	4 (57.1)	8 (88.9)	9 (75)	48 (69)	6 (75)	3 (60)	15 (65.2)	103 (62.4)
Female	20 (66.7)	3 (42.9)	1 (11.1)	3 (25)	23 (31)	2 (25)	2 (40)	8 (34.8)	62 (37.6)
Age (159)*									
<35	12 (40)	3 (42.9)	4 (50)	3 (25)	16 (23.2)		1 (20)	3 (15)	42 (26.4)
35–45	12 (40)	3 (42.9)	2 (25)	4 (33.3)	31 (44.9)	5 (62.5)	4 (80)	13 (65)	74 (46.5)
>45	6 (20)	1 (14.3)	2 (25)	5 (41.7)	22 (31.9)	3 (37.5)		4 (20)	43 (27)
Risk factor (153)*									
HTX	30 (100)	7 (100)	8 (88.9)	12 (100)	61 (95.3)	6 (75)	3 (75)	14 (73.7)	141 (92.2)
MSM					1 (1.6)	1 (12.5)	1 (25)	5 (26.3)	8 (5.2)
IVDU			1 (11.1)		2 (3.1)	1 (12.5)			4 (2.6)
Country of origin (163)*									
Spanish	11 (36.7)		4 (44.4)	1 (8.3)	6 (8.6)	4 (50)		10 (47.6)	36 (22)
East Europe	15 (50)	2 (28.6)	2 (22.2)					2 (9.5)	21 (12.9)
North Africa					3 (4.2)				3 (1.8)
West Africa	2 (6.7)	2 (28.6)	1 (11.1)	11 (91.7)	54 (76)	4 (50)	5 (100)	3 (14.3)	82 (50.3)
Central and South Africa	1 (3.3)	1(14.3)			8 (11.3)			2 (9.5)	12 (7.4)
Central and South America	1 (3.3)	2 (28.6)	2 (22.2)					4 (19)	9 (5.5)
Viral Load (158)*									
<10000	8 (26.7)	1 (14.3)	1 (12.5)	1 (8.3)	15 (22.1)	1 (12.5)		5 (23.8)	32 (20.3)
10000–100000	10 (33.3)	2 (28.6)	1 (12.5)	6 (50)	24 (35.3)	5 (62.5)	2 (50)	9 (42.9)	59 (37.3)
>100000	12 (40)	4 (57.1)	6 (75)	5 (41.7)	29 (42.6)	2 (25)	2 (50)	7 (33.3)	67 (42.4)
CD4 count (149)*									
<200	4 (16)		4 (50)	6 (50)	35 (52.2)	2 (25)	1 (20)	1 (5.3)	53 (35.6)
201–350	6 (24)	3 (60)	3 (37.5)	3 (25)	19 (28.4)	3 (37.5)	2 (40)	4 (21.1)	43 (28.9)
>350	15 (60)	2 (40)	1 (12.5)	3 (25)	13 (19.4)	3 (37.5)	2 (40)	14 (73.7)	53 (35.6)
**Sampling date interval**									
2005–2007	4 (13.3)	3 (42.9)		6 (50)	11 (15.5)	1 (12.5)		3 (13)	28 (17)
2007–2009	3 (10)	1 (14.3)	1 (11.1)	2 (16.7)	30 (42.3)	5 (62.5)	4 (80)	3 (13)	49 (29.7)
2009–2011	10 (33.3)	2 (28.6)	1 (11.1)	3 (25)	12 (16.9)	1 (12.5)	1 (20)	8 (34.8)	38 (23)
2011–2013	13 (43.3)	1 (14.3)	7 (77.8)	1 (8.3)	18 (25.4)	1 (12.5)		9 (39.1)	50 (30.3)
Total	30	7	9	12	71	8	5	23	165

*Date available for the number of indicated patients.

The multivariate logistic regression analyses demonstrated a higher risk of carrying HIV-1 subtype A for females (OR = 6.17, p = 0.026) and non Africans (OR = 0.08, p = 0.008; S3 Table). The other HIV-1 non-B genetic forms showed no predictive effect of the demographic, clinical and virological characteristics (data not shown).

**Fig 2:**
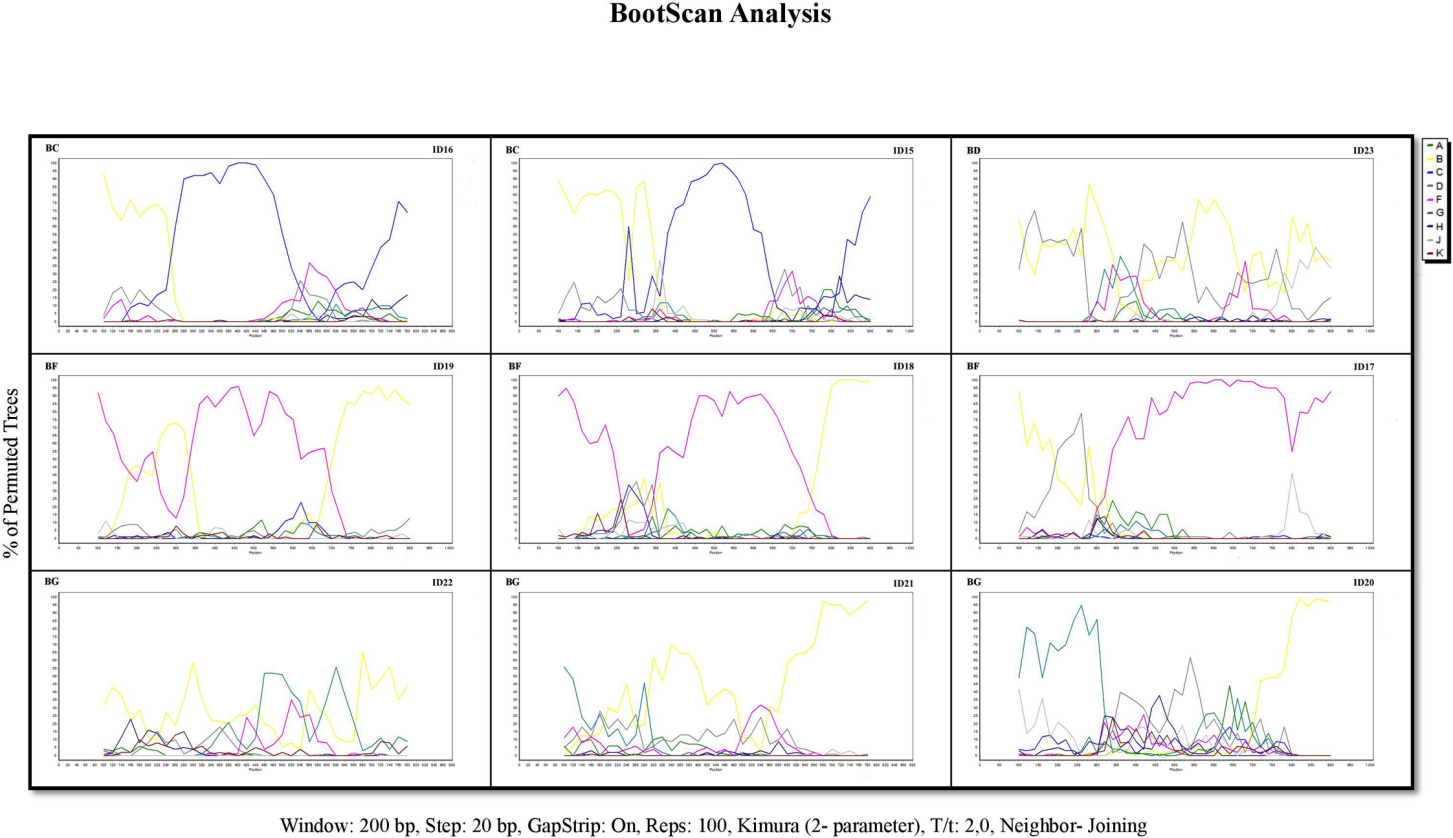
Bootscan analysis of the unique recombinant forms (URF) found in Eastern Andalusia. The analysis was applied to the concatenated sequences that corresponded to HXB2 coordinates 2283–2549 (PR) and 2661–3290 (RT).

Twenty-three patients were infected with unusual HIV-1 non-B variants (i.e., those variants found in four patients or fewer). Of them, 10 (44.4%) were observed in Spanish patients (Table 2). The recombination patterns for the different URFs obtained according to the Bootscan analysis are presented in Fig 2.

**Table 2:** Clinical, demographic and virological characteristics of the patients infected with infrequent HIV-1 non B genetic variants over the 2005–2012 period.

Patient ID	Genetic form	Gender (M/F)	Risk Factor	Age	Year of diagnosis	CD4 count	Viral Load	Country of origin
**1**	CRF01_AE	M	HTX	38	2008	764	421	Spain
**2**	CRF03_AB	M	MSM	28	2011	715	404000	Spain
**3**	CRF20_BG	M	HTX	65	2005	521	5910	Spain
**4**	CRF20_BG	F	HTX	40	2005	476	77700	Cuba
**5**	CRF22_01_A1	M	HTX	37	2009	474	11700	Equatorial Guinea
**6**	CRF25_CPX	F	UNK	NA	2012	NA	36300	Spain
**7**	CRF26_AU	F	HTX	25	2012	15	1180000	Spain
**8**	CRF37_CPX	M	HTX	39	2007	405	135000	Equatorial Guinea
**9**	CRF47_BF	M	HTX	39	2009	389	12279	Spain
**10**	CRF47_BF	F	HTX	32	2011	392	4122	Spain
**11**	CRF49_CPX	M	HTX	36	2009	288	2250000	Senegal
**12**	D	F	HTX	39	2011	443	22233	Equatorial Guinea
**13**	D	M	MSM	48	2009	526	55723	Spain
**14**	D	M	MSM	41	2009	416	417477	Colombia
**15**	URF BC	F	HTX	35	2011	723	27911	Romania
**16**	URF BC	M	HTX	40	2011	450	211933	Romania
**17**	URF BF	M	MSM	38	2011	246	143039	Spain
**18**	URF BF	F	HTX	38	2012	NA	44100	Brazil
**19**	URF BF	M	HTX	52	2006	462	3200	Argentina
**20**	URF BG	F	NA	NA	2008	NA	NA	NA
**21**	URF BG	M	NA	NA	2009	NA	NA	NA
**22**	URF BG	M	HTX	38	2009	NA	NA	Guinea-Bissau
**23**	URF BD	M	NA	49	2009	259	2800	Spain

NA: Not Available; M: Male; F: Female.

### Geographical distribution of the various HIV-1 non B genetic forms

**Fig 3:**
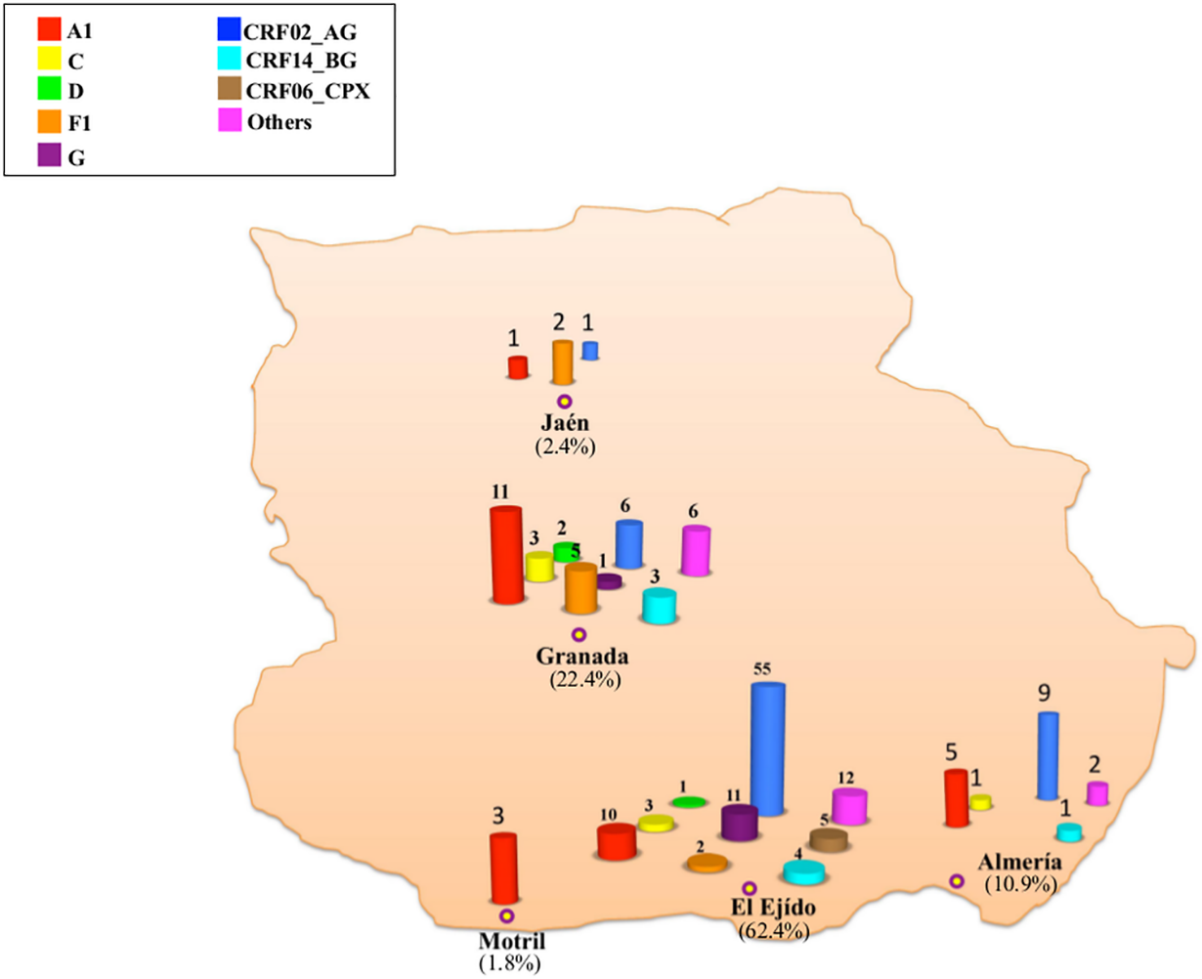
Geographical distribution of the patients infected with HIV-1 non-B variants over the 2005–2012 period. The percentage of each subtype/CRF in relation to all the HIV-1 non-B genetic forms is shown in each region.

The geographic distribution of the different HIV-1 non B subtypes and recombinant forms are represented on the map of Eastern Andalusia (Fig 3). Most of the patients infected with HIV-1 non-B variants were sampled in El Ejido (62.4%) or in the city of Granada, (22.4%), whereas non-B variants were less frequent in the cities of Almería (10.9%), Jaén (2.4%) and Motril (1.8%).

### Analysis of the putative geographical origins of the main HIV-1 non-B genetic forms found in Eastern Andalusia

**Fig 4:**
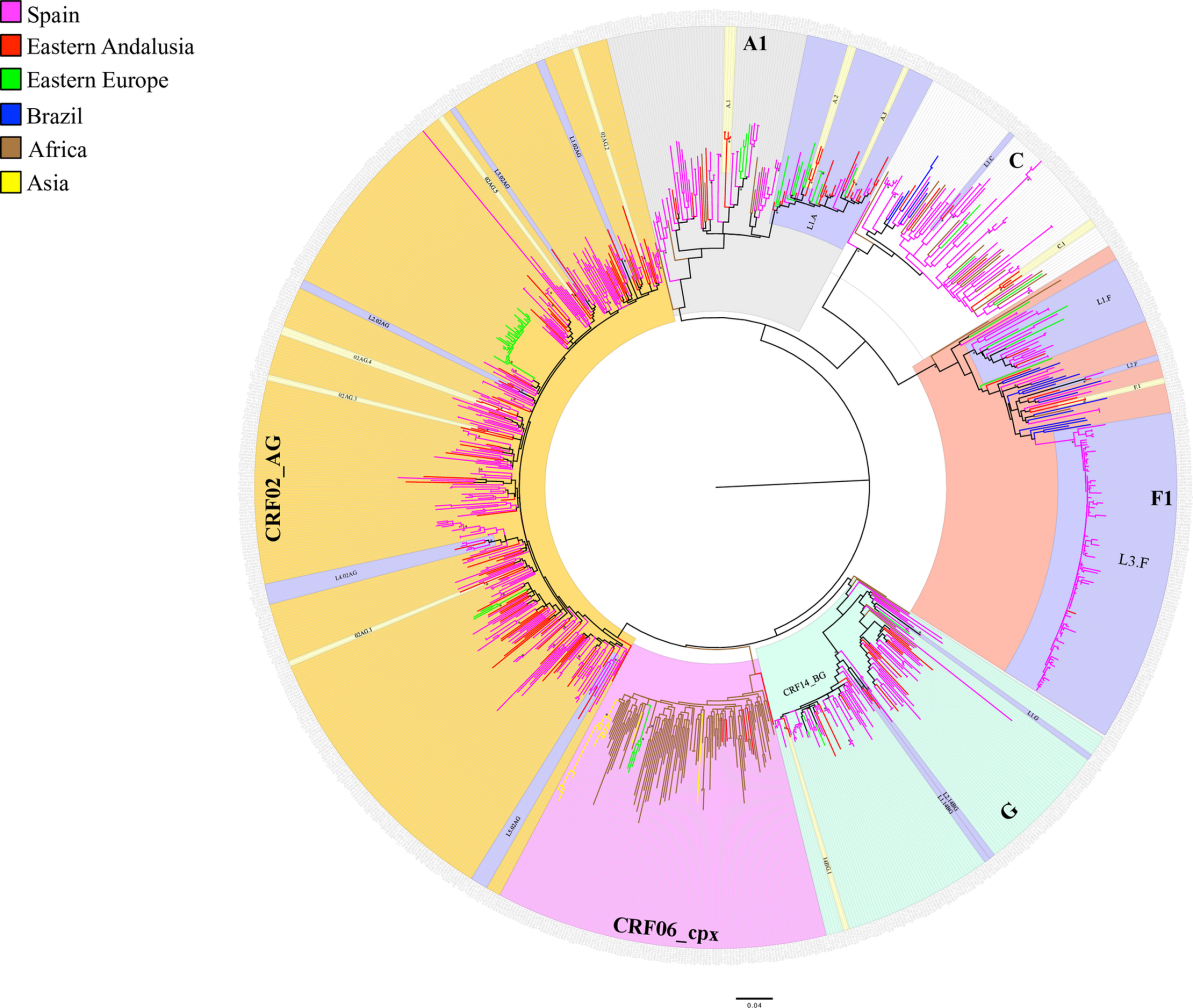
Global ML phylogenetic tree inferred for the main HIV-1 non-B genetic forms sampled in Eastern Andalusia. The phylogenetic tree was constructed by the general time-reversible with gamma-distributed rate heterogeneity across sites model of substitution implemented into RAxML. Branches are drawn on scale with the bar at the bottom, which represents 0.04 nucleotide substitution per site. Statistically highly supported nodes (bootstrap values >70%) are indicated by an asterisk (*). Andalusian clusters and international lineages are highlighted in yellow and blue, respectively. The Andalusian sequence names contain a three-part code: Sequence number, sampling site (AL: Almería, EJ: El Ejido GR: Granada, JA: Jaén, MO: Motril) and the code of the most likely country of infection.

In order to characterize the phylogenetic relationship of the patients infected with the most frequently found HIV-1 non-B variants (those found in ≥ 5 patients), the global ML tree (Fig 4) revealed the existence of 13 international lineages in Eastern Andalusia (Table 3) and 11 Andalusian clusters (Table 4) that involved patients in our cohort.

**Table 3:** HIV-1 non-B international lineages involving sequences sampled in Eastern Andalusia and sequences from different countries.

Lineages	Sequences (EA;OR)	Country of origin of the patients sampled in our cohort (n)	Node support (BT)	Country of collection for other sequences (n)	Most likely countries of infection for other patients (Accession ID)	Articles with related sequences
Subtype A						
**L1.A**	21;23	Spanish (5), Russian (14), Lithuanian (1) Dominican (1)	97	Spain (11) Bulgaria (10) Poland (1) Croatia (1)	Ukraine (KC340416) Russia (KC340417, KC340407, KC340486, KC340436, KC340495)	[20,38,39]
Subtype C						
**L1.C**	1;1	Senegalese (1)	100	Spain (1)	Morocco (JQ351995)	[40]
Subtype F						
**L1.F**	2:21	Romanian (2)	70	Spain (19) Bulgaria (2)	Romania (KC340133, KC340134, KC340419, KC340378, KC340643, KC340132)	[20,39,41]
**L2.F**	1;1	Spanish (1)	100	Spain (1)	Sub-Saharan Africa (KC340440)	
**L3.F**	1;112	Spanish (1)	100	Spain (112)	Spain (KJ883085-KJ883089, KJ883091-KJ883108, KJ883110-KJ883152, JN010216-JN859590)	[42,43]
Subtype G						
**L1.G**	1;1	Nigerian (1)	100	Spain (1)	Nigeria (FJ481667)	[44]
CRF_14BG						
**L1.14BG**	1;1	Guinean-Bissau (1)	100	Spain (1)	Equatorial-Guinea (JX428555)	[45]
**L2.14BG**	1;1	Guinean (1)	100	Spain (1)	Equatorial-Guinea (EU255306)	[45]
Form CRF02_AG						
**L1.02AG**	1;2	Guinean (1)	100	Spain (2)	Ghana (KC340379)	[23]
**L2.02AG**	2;1	Moroccan (1) Spanish (1)	86	Spain (1)	Cameroon (KC340644)	
**L3.02AG**	1;1	Nigerian (1)	86	Spain (1)	Spain (HF567877)	[46]
**L4.02AG**	1;6	Ghanaian (1)	96	Spain (6)	Bolivia (EU255444) Ecuador (KC340123, KC340124, FJ481711)	[44,47]
**L5.02AG**	1;5	Equatorial-Guinean (1)	82	Spain (5)	Equatorial-Guinea (EU255527)	[47,48]

EA: sequences sampled in Eastern Andalusia; OR: sequences sampled in other geographic regions; BT: bootstrap (in the global maximum-likelihood tree).

**Table 4:** Demographical, clinical, virological and phylogenetic characteristics of the Andalusian clusters found for the main HIV-1 non B variants.

Cluster ID	No. Patients	Sampling interval	Support (BT:PP)	Risk factor	Country of origin	Viral Load (median, IQR) Log_10_	CD4 count (median, IQR)	Location area	tMRCA (95% HPD)	
**Subtype A**										
A.1	4	2011	100;0.9	HTX	Spanish	5 (4.6–6)	590 (534–701)	Granada	2008.5 (2006.6–2010.3)	
A.2	3	2007–2011	96;0.9	HTX	Spanish-Russian	4.7 (4.4–5.3)	301 (152–517)	Granada-El Ejido	2003.9 (1998.4–2004.7)	
A.3	2	2009–2010	100;0.9	HTX	Spanish-Russian	4.9 (4–5)	391 (325–456)	El Ejido	2008 (2007.4–2009)	
**Subtype C**										
C.1	3	2005–2012	100;0.9	HTX	Romanian-Brazilian	5.2 (5.1–5.3)	499 (253–739)	Granada-Ejido	1998.3 (1992.3–2001.9)	
**Subtype F**										
F.	2	2011–2012	90;0.9	HTX	Brazilian	5.7(5.4–5.9)	62 (50–73)	Jaén	2010.2 (2010–2011)	
**Form CRF14_BG**									
14BG.1	2	2005–2007	100;0.9	HTX	Spanish	5	425 (267–583)	Granada	2004.4 (2003.8–2005)	
**Form CRF02AG**											
02AG.1	2	2007	100;0.9	HTX	Guinean	5.6(4–6)	309 (180–438)	El Ejido	2003.8 (2001–2006)	
02AG.2	2	2007–2010	100;0.9	HTX	Senegalese	3(4–5)	391 (381–400)	El Ejido	2006.4 (2006–2007)	
02AG.3	2	2011	99;0.9	HTX	Spanish-Moroccan	5.9(3–6)	326 (100–551)	Granada-Almería	2008.2 (2007.2–2009.9)	
02AG.4	3	2006–2011	98;0.9	HTX	Spanish- Mali-	5.5(5.3–6.2)	140 (78–306)	El Ejido	2003.6 (2000.3–2005.3)	
02AG.5	2	2008	100;0.9	HTX	Ghana	6	126 (45–200)	El Ejido	2007.7 (2007.2–2009)	

**Fig 5:**
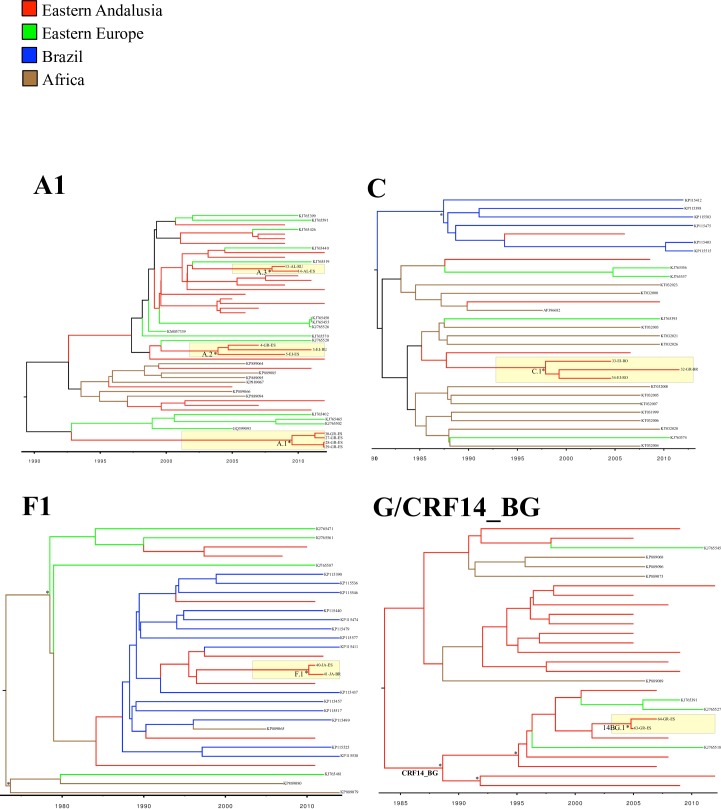
Bayesian phylogenetic tree inferred for the subtype A1, C, F1 and G/CRF14_BG *pol* sequences sampled in Eastern Andalusia and genetically similar sequences from GenBank. Red branches correspond to the sequences sampled in eastern Andalusia from 2005 to 2012. Statistically highly supported nodes (posterior probability values above 0.9) are indicated with an asterisk (*). Andalusian clusters are highlighted in yellow. Andalusian sequences names contain a three-part code: Sequence number, sampling site (AL: Almería, EJ: El Ejido GR: Granada, JA: Jaén, MO: Motril) and the code of the most likely country of infection.

**Fig 6:**
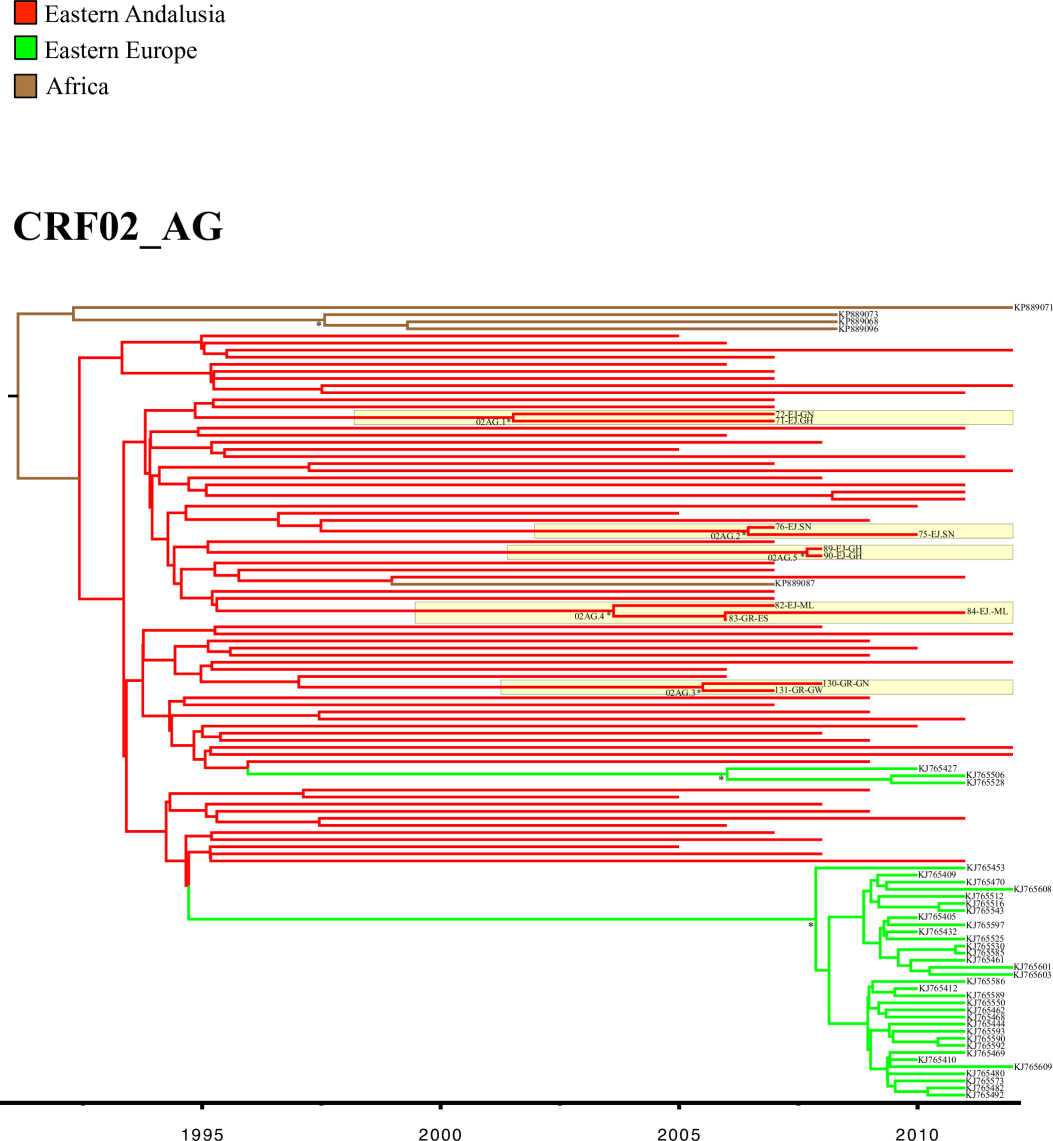
Bayesian phylogenetic tree inferred for the CRF02_AG *pol* sequences sampled in Eastern Andalusia and genetically similar sequences from GenBank. Red branches correspond to the sequences sampled in eastern Andalusia from 2005 to 2012. Statistically highly supported nodes (posterior probability values above 0.9) are indicated with an asterisk (*). Andalusian clusters are highlighted in yellow. Andalusian sequences names contain a three-part code: Sequence number, sampling site (AL: Almería, EJ: El Ejido GR: Granada, JA: Jaén, MO: Motril) and the code of the most likely country of infection.

The Bayesian analyses (Figs 5 and 6) showed that most of these Andalusian clusters originated in the first decade of this century, and mainly included patients sampled in El Ejido. The low CD4 count of the patients included in most of these transmission networks suggests a late HIV diagnosis in a high proportion of patients (Table 4).

In order to provide more information about the scale of the trees shown, we provide in the S4 Table the distribution of patristic (uncorrected) pairwise genetic distances between sequences included in each of the ML and Bayesian trees generated in this article.

### Non-recombinant subtypes

Thirty (18.2%) patients were infected with HIV-1 subtype A1. The viral sequences were genetically similar according to HIV-BLAST to 21 GenBank sequences from Bulgaria, the Democratic Republic of Congo, Croatia and Greece with 13, 6, 1 and 1 cases, respectively. The ML analysis (Fig 4) detected a large international lineage that involved sequences from Eastern Europe (lineage L1.A1 in Table 3) and grouped 21 patients from our cohort: 16 women born abroad (Eastern Europe (n = 14), the Dominican Republic (n = 1) and Lithuania (n = 1)) and 5 Spanish men. This lineage also included 23 GenBank sequences, also originating from Eastern Europe: Bulgaria, n = 10, Russia, n = 5, Poland, n = 1 and the Ukraine, n = 1. Within this lineage, we found two clusters (A.1 and A.2), formed exclusively by Spanish men and female sex workers born in Russia, all being patients sampled in Eastern Andalusia. The A.1 local cluster involved 4 sequences from Spanish patients living in the capital of Granada, its origin was estimated to be 2008.5 (95%CI: 2006.6–2010.3), and the sequences presented the resistance mutation K103N in the RT gene. This cluster was also phylogenetically related to viruses that circulate in Eastern Europe. Unlike most of the HIV-1 non-B clusters, patients in the A.1 cluster showed a high CD4 count (mean = 590, range = 534–701). Moreover, the Bayesian phylogenetic tree revealed short internode branches, which may indicate short times between infections. Finally, five subtype A1 sequences from our cohort corresponded to patients from Africa: Mali, n = 2, Equatorial Guinea, n = 1 and Spain, n = 2, not clustered in transmission cluster.

Seven (4.2%) sequences corresponded to HIV-1 subtype C, and showed high genetic similarity to 23 GenBank sequences sampled in South Africa (n = 12), Brazil (n = 6) and Bulgaria (n = 4). We thus found two main ways of subtype C entrance to our area: South Africa and Brazil: a Brazilian male patient from our cohort grouped with 6 GenBank sequences from Brazil; and a South African male patient grouped with GenBank sequences from South Africa (n = 2) and Somalia (n = 1) (Fig 4). Within this subtype, we also found a single cluster (C.1) formed by patients from Brazil (n = 1) and Romania (n = 2).

Nine (5.5%) sequences corresponded to HIV-1 subtype F1 and showed a high genetic similarity to 21 GenBank sequences from Brazil (n = 14), Bulgaria (n = 4) and the Democratic Republic of Congo (n = 3). We found only one Andalusian F1 cluster: a sequence pair (cluster F.1), that originated in 2010.2 (95%CI: 2010–2011) and was formed by two male injection drug users sampled in Jaen and who were of Brazilian and Spanish origins. This sequence pair was included among GenBank sequences from Brazil in the ML tree. However, we found 2 international lineages: L1.F, which grouped two Romanian heterosexual patients from our cohort with GenBank sequences sampled in Eastern Europe, mainly Romania (n = 6) and Bulgaria (n = 2). The second F1 subtype lineage (L3.F) included Spanish men who have sex with men (MSM) sampled in North Spain, and also a MSM from our cohort.

Twelve (7.3%) patients of our cohort were infected with HIV-1 subtype G, who came from different western and central African countries: Mali (n = 1), Nigeria (n = 6), Ghana (n = 3) and Guinea-Bissau (n = 1). They presented high genetic similarity to 5 GenBank sequences from the Republic of Congo (n = 4) and Bulgaria (n = 1). None of these sequences was epidemiologically related according to our data. We found only one Nigerian patient whose sequence grouped with another one of the same country of origin (lineage L1.G).

### HIV-1 recombinant forms

Eight (4.8%) patients in our cohort were infected with the recombinant CRF14_BG form, which in the *pol* analyses typically forms a monophyletic cluster within the subtype G crown. These eight patients came from Spain (n = 4), Guinea (n = 2), and Guinea-Bissau (n = 2). We also found a single small Andalusian cluster (cluster 14BG.1), which originated in 2004.4 (95% CI:2003.8–2005), and was formed by two Spanish patients. Finally, two patients from Guinea and Guinea Bissau grouped with sequences from Equatorial Guinea (lineages L1.14BG and L2.14BG).

We found 71 (43%) patients, mainly from western African countries (77.5%), infected with CRF02_AG. Of these, 11 (14%) were grouped into five small Andalusian Cluster: 4 clusters of two patients and one with three patients. We detected 5 different lineages (L1.02AG-L5.02AG) of viruses sampled in other countries, with patients from our cohort who came mainly from Western Africa.

To study the phylogenetic profile of variant CRF06_cpx, we used all the sequences available in Los Alamos HIV given their small number, n = 110 (see Fig 4). We found 5 patients in our cohort (3%) to be infected with variant CRF06_cpx, who came from different western African countries: Nigeria (n = 3), Ghana (n = 1) and Senegal (n = 1). These sequences grouped with GenBank sequences from the neighboring Western African countries of Burkina Faso, Togo and Nigeria. However, we found no significant association among the patients infected with this genetic form, and the CRF06_cpx sequences sampled in our cohort were interspersed in the tree.

## Discussion

In Eastern Andalusia, most HIV-1 non-B subtype genetic forms were found among immigrant heterosexual population, mainly African males or Eastern European females. These patients were living preferentially in El Ejido, an area that potentially acts as a gateway for diverse HIV-1 variants to enter the Eastern Andalusian region. These findings are explained by the fact the El Ejido’s economy is mainly based on greenhouse farming, for which a large industry has emerged in recent years thanks to immigrant labor, made up of people mainly from Africa.

The prevalence of HIV-1 non-B variants in eastern Andalusia is similar to that reported in a study performed in the nearby Western areas of Andalusia (≈23%) [49], but is still much higher than that found elsewhere in Spain [6,7]. An increased prevalence has been noted for HIV-1 non-B variants and their genetic diversity in Eastern Andalusia in recent years: 22% of autochthonous patients were infected with HIV-1 non-B forms between 2005 and 2012, as opposed to the 12.8% reported in former studies conducted between 1997 and 2001 [50]. We also detected 12 different CRFs and nine URFs, a variability that is probably related to the increased migration rate reported in southern Spain in the last decade [49,51].

The least frequent HIV-1 non-B variants were detected often among Spanish patients (43%, [10/23]), and most of the clusters formed by these variants included at least one Spanish patient (55%, [6/11]). These data suggest that although these HIV-1 non-B variants seem to be due to imported cases in most cases, they have also gradually penetrated the autochthonous population in recent years.

The phylogenetic and epidemiological study of the HIV-1 non-B variants in our region showed that these variants account for high proportion of infections among migrant patients, and that these viruses were genetically close to those circulating in these subjects’ countries of origin. This indicates that many patients were infected before they arrived in Spain. These sequences sampled in other countries, and available in public databases, act as a control to avoid overestimating the local transmission clusters that include patients who are most probably unrelated in epidemiological terms.

As previously shown in a national study [7], CRF02_AG was the most frequent HIV non-B variant in our population (43%). Nonetheless, the small proportion of their phylogenetic association is surprising (14%, [11/71]). This clustering rate was much higher for other HIV-1 non-B subtypes, such as subtype A1 (27.3%, [9/33]), where we discovered an international lineage (L1.A1) that mostly included a particularly vulnerable group of Russian female sex workers and potentially their local customers.

According to our analysis, it would appear that most of the non-B cases detected in Eastern Andalusia were generally imported cases as most were identified in immigrant populations. Our analyses suggest that many of these cases form part of international HIV-1 lineages that originated in Eastern Europe, South America and sub-Saharan Africa. However, we also identified 11 intra-region clusters, which might suggest the local dissemination of some non-B variants, particularly those which involve autochthonous Spanish subjects (6/11) and recent emergence times according to the phylogenetic reconstruction. On the other hand, clusters formed by foreign subjects with old common ancestors most likely reflect imported infections.

The methods used herein involve a number of sampling limitations that affect this and many other similar studies. Since we relied on a BLAST search to identify the genetically closest sequences (from both Spain and abroad) that could form part of the same transmission networks as our sequences, we depended on the sequences deposited in databases. Unfortunately, this availability is sometimes very low, particularly for non-B variants. Therefore, we cannot rule out that close and more informative sequences were not captured as they have not been sampled. This was the reason why we added all the sequences available in HIV Los Alamos collected in Spain. We also demonstrated the presence of 13 different lineages of viruses that circulated in our region, which grouped with other patients from different Spanish cohorts, mainly foreign patients.

Fortunately, very few sequences included in transmission clusters persented resistance to first-line antiretroviral drugs. This information agrees with the common conception that viruses with resistance mutations present a biological disadvantage against wild strains, which weakens their transmission efficacy. Likewise, the drug resistance mutations detected affect mainly reverse transcriptase inhibitor drugs. We detected transmitted resistance mutations in four of the five patients grouped in Cluster A.4, which would cause high level resistance to nevirapine and efavirenz. We decided to study only the resistance mutations present in transmission clusters, which would have a stronger epidemiological impact. Further detailed information will be provided in future works.

The constant epidemiological surveillance in our population, for which phylogenetic analysis tools are used, is a particularly important measure to study past outbreaks of genetic HIV-1 non-B subtype variants, and to prevent future ones. Likewise, as transmission cluster size seems to predict its expansion in time [52], we could expect some transmission chains of HIV-1 non-subtype variants to become larger in size in forthcoming years, and more Spanish individuals to be included. We herein detected the presence of one patient from our cohort related to a fast spreading cluster among Spanish MSM infected with subtype F in Galicia (NW Spain) [43], a transmission cluster which, as Delgado *et al*. suggest, would probably be closely linked to viruses that circulate in Eastern Europe [42]. These authors [53] have also described a subtype A cluster that is being transmitted among individuals in different areas of Spain. Finally, Patiño *et al*. [54] have warned about the novel appearance of variant CRF19_cpx among Spanish MSM individuals.

Adequate knowledge about the characteristics of local epidemics, the study of risk groups and the prevalence of different viral subtypes are all fundamental aspects to successfully design HIV-1 prevention campaigns. In the present study, we demonstrate that phylogenetic studies which combine demographic, clinical and geographical data from different HIV-1 non-B subtypes in Eastern Andalusia provide very useful information to epidemiologically monitor and control HIV-1 spread and its origin in imported cases. Its use will help to reinforce and implement efficient actions to prevent HIV-1 from spreading between autochthonous and migrant populations.

## Supporting information

S1 Table HIV-1 reference sequence dataset used in the phylogenetic analysis. (DOCX)

S2 Table Evolutionary rates for each of the main HIV-1 lineages found in this study obtained through Bayesian phylogenetic inference. (DOCX)

S3 Table Multivariate logistic regression analysis performed for the HIV-1 subtype A1 infections. (DOCX)

S4 Table Distribution of patristic (uncorrected) genetic distances among the HIV-1 *pol* sequences in each dataset included in this study. (DOCX)

## References

[1] Vidal N, Mulanga C, Bazepeo SE, Mwamba JK, Tshimpaka J-W, Kashi M, et al Distribution of HIV-1 variants in the Democratic Republic of Congo suggests increase of subtype C in Kinshasa between 1997 and 2002. J Acquir Immune Defic Syndr 1999. 2005 12 1;40(4):456–62. 10.1097/01.qai.0000159670.18326.94 16280702

[2] Taniguchi Y, Takehisa J, Bikandou B, Mboudjeka I, N’Doundou-N’Kodia M-Y, Obengui null, et al Genetic subtypes of HIV type 1 based on the vpu/env sequences in the Republic of Congo. AIDS Res Hum Retroviruses. 2002 1 1;18(1):79–83. doi: 10.1089/088922202753394745 11804559 10.1089/088922202753394745

[3] Shankarappa R, Margolick JB, Gange SJ, Rodrigo AG, Upchurch D, Farzadegan H, et al Consistent viral evolutionary changes associated with the progression of human immunodeficiency virus type 1 infection. J Virol. 1999 12;73(12):10489–502. 10559367 10.1128/jvi.73.12.10489-10502.1999 PMC113104

[4] Robertson DL, Anderson JP, Bradac JA, Carr JK, Foley B, Funkhouser RK, et al HIV-1 nomenclature proposal. Science. 2000 4 7;288(5463):55–6. 10766634 10.1126/science.288.5463.55d

[5] Hemelaar J, Gouws E, Ghys PD, Osmanov S, WHO-UNAIDS Network for HIV Isolation and Characterisation. Global trends in molecular epidemiology of HIV-1 during 2000–2007. AIDS Lond Engl. 2011 3 13;25(5):679–89. 10.1097/QAD.0b013e328342ff93 PMC3755761 21297424

[6] Yebra G, de Mulder M, Martín L, Rodríguez C, Labarga P, Viciana I, et al Most HIV type 1 non-B infections in the Spanish cohort of antiretroviral treatment-naïve HIV-infected patients (CoRIS) are due to recombinant viruses. J Clin Microbiol. 2012 2;50(2):407–13. doi: 10.1128/JCM.05798-11 22162552 10.1128/JCM.05798-11 PMC3264155

[7] García F, Pérez-Cachafeiro S, Guillot V, Alvarez M, Pérez-Romero P, Pérez-Elías MJ, et al Transmission of HIV drug resistance and non-B subtype distribution in the Spanish cohort of antiretroviral treatment naïve HIV-infected individuals (CoRIS). Antiviral Res. 2011 8;91(2):150–3. doi: 10.1016/j.antiviral.2011.05.010 21663768 10.1016/j.antiviral.2011.05.010

[8] Artenstein AW, VanCott TC, Mascola JR, Carr JK, Hegerich PA, Gaywee J, et al Dual infection with human immunodeficiency virus type 1 of distinct envelope subtypes in humans. J Infect Dis. 1995 4;171(4):805–10. 7706806 10.1093/infdis/171.4.805

[9] van Harmelen J, Wood R, Lambrick M, Rybicki EP, Williamson AL, Williamson C. An association between HIV-1 subtypes and mode of transmission in Cape Town, South Africa. AIDS Lond Engl. 1997 1;11(1):81–7. 10.1097/00002030-199701000-00012 9110079

[10] Tscherning C, Alaeus A, Fredriksson R, Björndal A, Deng H, Littman DR, et al Differences in chemokine coreceptor usage between genetic subtypes of HIV-1. Virology. 1998 2 15;241(2):181–8. doi: 10.1006/viro.1997.8980 9499793 10.1006/viro.1997.8980

[11] Kanki PJ, Hamel DJ, Sankalé JL, Hsieh C c, Thior I, Barin F, et al Human immunodeficiency virus type 1 subtypes differ in disease progression. J Infect Dis. 1999 1;179(1):68–73. doi: 10.1086/314557 9841824 10.1086/314557

[12] Apetrei C, Descamps D, Collin G, Loussert-Ajaka I, Damond F, Duca M, et al Human immunodeficiency virus type 1 subtype F reverse transcriptase sequence and drug susceptibility. J Virol. 1998 5;72(5):3534–8. 9557632 10.1128/jvi.72.5.3534-3538.1998 PMC109572

[13] Taylor BS, Sobieszczyk ME, McCutchan FE, Hammer SM. The challenge of HIV-1 subtype diversity. N Engl J Med. 2008 4 10;358(15):1590–602. doi: 10.1056/NEJMra0706737 18403767 10.1056/NEJMra0706737 PMC2614444

[14] Alaeus A, Lidman K, Sönnerborg A, Albert J. Subtype-specific problems with quantification of plasma HIV-1 RNA. AIDS Lond Engl. 1997 6;11(7):859–65. 10.1097/00002030-199707000-00004 9189210

[15] Parekh B, Phillips S, Granade TC, Baggs J, Hu DJ, Respess R. Impact of HIV type 1 subtype variation on viral RNA quantitation. AIDS Res Hum Retroviruses. 1999 1 20;15(2):133–42. doi: 10.1089/088922299311556 10029245 10.1089/088922299311556

[16] Brennan CA, Bodelle P, Coffey R, Harris B, Holzmayer V, Luk K-C, et al HIV global surveillance: foundation for retroviral discovery and assay development. J Med Virol. 2006;78 Suppl 1:S24–9. 16622874 10.1002/jmv.20603

[17] Gilbert MTP, Rambaut A, Wlasiuk G, Spira TJ, Pitchenik AE, Worobey M. The emergence of HIV/AIDS in the Americas and beyond. Proc Natl Acad Sci U S A. 2007 11 20;104(47):18566–70. doi: 10.1073/pnas.0705329104 17978186 10.1073/pnas.0705329104 PMC2141817

[18] Gray RR, Tatem AJ, Lamers S, Hou W, Laeyendecker O, Serwadda D, et al Spatial phylodynamics of HIV-1 epidemic emergence in east Africa. AIDS Lond Engl. 2009 9 10;23(14):F9–17. 10.1097/QAD.0b013e32832faf61 PMC2742553 19644346

[19] Esbjörnsson J, Mild M, Månsson F, Norrgren H, Medstrand P. HIV-1 molecular epidemiology in Guinea-Bissau, West Africa: origin, demography and migrations. PloS One. 2011;6(2):e17025 doi: 10.1371/journal.pone.0017025 21365013 10.1371/journal.pone.0017025 PMC3041826

[20] González-Alba JM, Holguín Á, Garcia R, García-Bujalance S, Alonso R, Suárez A, et al Molecular Surveillance of HIV-1 in Madrid, Spain: a Phylogeographic Analysis ▿. J Virol. 2011 10;85(20):10755–63. doi: 10.1128/JVI.00454-11 21795343 10.1128/JVI.00454-11 PMC3187488

[21] Aldous JL, Pond SK, Poon A, Jain S, Qin H, Kahn JS, et al Characterizing HIV transmission networks across the United States. Clin Infect Dis Off Publ Infect Dis Soc Am. 2012 10;55(8):1135–43. 10.1093/cid/cis612 PMC3529609 22784872

[22] Grabowski MK, Redd AD. Molecular tools for studying HIV transmission in sexual networks. Curr Opin HIV AIDS. 2014 3;9(2):126–33. doi: 10.1097/COH.0000000000000040 24384502 10.1097/COH.0000000000000040 PMC4109889

[23] Cuevas MT, Muñoz-Nieto M, Thomson MM, Delgado E, Iribarren JA, Cilla G, et al HIV-1 Transmission Cluster With T215D Revertant Mutation Among Newly Diagnosed Patients From the Basque Country, Spain: JAIDS J Acquir Immune Defic Syndr. 2009 5;51(1):99–103. doi: 10.1097/QAI.0b013e318199063e 19282784 10.1097/QAI.0b013e318199063e

[24] Vega Y, Delgado E, Fernández-García A, Cuevas MT, Thomson MM, Montero V, et al Epidemiological Surveillance of HIV-1 Transmitted Drug Resistance in Spain in 2004–2012: Relevance of Transmission Clusters in the Propagation of Resistance Mutations. PLOS ONE. 2015 5 26;10(5):e0125699 doi: 10.1371/journal.pone.0125699 26010948 10.1371/journal.pone.0125699 PMC4444345

[25] Pérez-Parra S, Chueca-Porcuna N, Álvarez-Estevez M, Pasquau J, Omar M, Collado A, et al [Study of human immunodeficiency virus transmission chains in Andalusia: Analysis from baseline antiretroviral resistance sequences.]. Enferm Infecc Microbiol Clin. 2015 1 31; 10.1016/j.eimc.2014.11.016 25648468

[26] Larkin MA, Blackshields G, Brown NP, Chenna R, McGettigan PA, McWilliam H, et al Clustal W and Clustal X version 2.0. Bioinforma Oxf Engl. 2007 11 1;23(21):2947–8. 10.1093/bioinformatics/btm404 17846036

[27] Miller MA, Pfeiffer W, Schwartz T. Creating the CIPRES Science Gateway for inference of large phylogenetic trees. In: Gateway Computing Environments Workshop (GCE), 2010. 2010. p. 1–8.

[28] Hillis DM, Bull JJ. An Empirical Test of Bootstrapping as a Method for Assessing Confidence in Phylogenetic Analysis. Syst Biol. 1993 6 1;42(2):182–92.

[29] Lole KS, Bollinger RC, Paranjape RS, Gadkari D, Kulkarni SS, Novak NG, et al Full-length human immunodeficiency virus type 1 genomes from subtype C-infected seroconverters in India, with evidence of intersubtype recombination. J Virol. 1999 1;73(1):152–60. 9847317 10.1128/jvi.73.1.152-160.1999 PMC103818

[30] Leigh Brown AJ, Lycett SJ, Weinert L, Hughes GJ, Fearnhill E, Dunn DT, et al Transmission network parameters estimated from HIV sequences for a nationwide epidemic. J Infect Dis. 2011 11;204(9):1463–9. doi: 10.1093/infdis/jir550 21921202 10.1093/infdis/jir550 PMC3182313

[31] Kouyos RD, von Wyl V, Yerly S, Böni J, Taffé P, Shah C, et al Molecular epidemiology reveals long-term changes in HIV type 1 subtype B transmission in Switzerland. J Infect Dis. 2010 5 15;201(10):1488–97. doi: 10.1086/651951 20384495 10.1086/651951

[32] Esbjörnsson J, Mild M, Audelin A, Fonager J, Skar H, Bruun Jørgensen L, et al HIV-1 transmission between MSM and heterosexuals, and increasing proportions of circulating recombinant forms in the Nordic Countries. Virus Evol. 2016 1;2(1):vew010 doi: 10.1093/ve/vew010 27774303 10.1093/ve/vew010 PMC4989887

[33] Drummond AJ, Suchard MA, Xie D, Rambaut A. Bayesian phylogenetics with BEAUti and the BEAST 1.7. Mol Biol Evol. 2012 8;29(8):1969–73. doi: 10.1093/molbev/mss075 22367748 10.1093/molbev/mss075 PMC3408070

[34] Drummond AJ, Ho SYW, Phillips MJ, Rambaut A. Relaxed Phylogenetics and Dating with Confidence. PLoS Biol. 2006 3 14;4(5):e88 doi: 10.1371/journal.pbio.0040088 16683862 10.1371/journal.pbio.0040088 PMC1395354

[35] Drummond AJ, Rambaut A, Shapiro B, Pybus OG. Bayesian coalescent inference of past population dynamics from molecular sequences. Mol Biol Evol. 2005 5;22(5):1185–92. doi: 10.1093/molbev/msi103 15703244 10.1093/molbev/msi103

[36] Hué S, Brown AE, Ragonnet-Cronin M, Lycett SJ, Dunn DT, Fearnhill E, et al Phylogenetic analyses reveal HIV-1 infections between men misclassified as heterosexual transmissions. AIDS Lond Engl. 2014 8 24;28(13):1967–75. 10.1097/QAD.0000000000000383 24991999

[37] Bennett DE, Camacho RJ, Otelea D, Kuritzkes DR, Fleury H, Kiuchi M, et al Drug Resistance Mutations for Surveillance of Transmitted HIV-1 Drug-Resistance: 2009 Update. PLoS ONE [Internet]. 2009 3 6 [cited 2016 Sep 3];4(3). Available from: http://www.ncbi.nlm.nih.gov/pmc/articles/PMC2648874/10.1371/journal.pone.0004724 PMC2648874 19266092

[38] Alexiev I, Shankar A, Wensing AMJ, Beshkov D, Elenkov I, Stoycheva M, et al Low HIV-1 transmitted drug resistance in Bulgaria against a background of high clade diversity. J Antimicrob Chemother. 2015;70(6):1874–80. doi: 10.1093/jac/dkv011 25652746 10.1093/jac/dkv011 PMC11292601

[39] De Mendoza C, Garrido C, Poveda E, Corral A, Zahonero N, Treviño A, et al Changes in drug resistance patterns following the introduction of HIV type 1 non-B subtypes in Spain. AIDS Res Hum Retroviruses. 2009 10;25(10):967–72. doi: 10.1089/aid.2008.0166 19842792 10.1089/aid.2008.0166

[40] Trends in Drug Resistance Prevalence in HIV-1–infected Children in Madrid (PDF Download Available) [Internet]. ResearchGate. [cited 2017 Mar 15]. Available from: https://www.researchgate.net/publication/229074263_Trends_in_Drug_Resistance_Prevalence_in_HIV-1-infected_Children_in_Madrid

[41] Fernández-García A, Cuevas MT, Vinogradova A, Rakhmanova A, Pérez-Alvarez L, de Castro RO, et al Near full-length genome characterization of a newly identified HIV type 1 subtype F variant circulating in St. Petersburg, Russia. AIDS Res Hum Retroviruses. 2009 11;25(11):1187–91. doi: 10.1089/aid.2009.0140 19943791 10.1089/aid.2009.0140

[42] Delgado E, Cuevas MT, Domínguez F, Vega Y, Cabello M, Fernández-García A, et al Phylogeny and Phylogeography of a Recent HIV-1 Subtype F Outbreak among Men Who Have Sex with Men in Spain Deriving from a Cluster with a Wide Geographic Circulation in Western Europe. PloS One. 2015;10(11):e0143325 doi: 10.1371/journal.pone.0143325 26599410 10.1371/journal.pone.0143325 PMC4658047

[43] Thomson MM, Fernández-García A, Delgado E, Vega Y, Díez-Fuertes F, Sánchez-Martínez M, et al Rapid expansion of a HIV-1 subtype F cluster of recent origin among men who have sex with men in Galicia, Spain. J Acquir Immune Defic Syndr 1999. 2012 3 1;59(3):e49–51. 10.1097/QAI.0b013e3182400fc4 22327248

[44] Yebra G, de Mulder M, del Romero J, Rodríguez C, Holguín A. HIV-1 non-B subtypes: High transmitted NNRTI-resistance in Spain and impaired genotypic resistance interpretation due to variability. Antiviral Res. 2010 2;85(2):409–17. doi: 10.1016/j.antiviral.2009.11.010 20004217 10.1016/j.antiviral.2009.11.010

[45] Yebra G, de Mulder M, Pérez-Elías MJ, Pérez-Molina JA, Galán JC, Llenas-García J, et al Increase of transmitted drug resistance among HIV-infected sub-Saharan Africans residing in Spain in contrast to the native population. PloS One. 2011;6(10):e26757 doi: 10.1371/journal.pone.0026757 22046345 10.1371/journal.pone.0026757 PMC3201965

[46] Bracho MA, Sentandreu V, Alastrué I, Belda J, Juan A, Fernández-García E, et al Emerging trends in CRF02_AG variants transmission among men who have sex with men in Spain. J Acquir Immune Defic Syndr 1999. 2014 3 1;65(3):e130–3. 10.1097/01.qai.0000435602.73469.56 24091696

[47] Holguín A, de Mulder M, Yebra G, López M, Soriano V. Increase of non-B subtypes and recombinants among newly diagnosed HIV-1 native Spaniards and immigrants in Spain. Curr HIV Res. 2008 6;6(4):327–34. 18691031 10.2174/157016208785132455

[48] Fernández-García A, Cuevas MT, Muñoz-Nieto M, Ocampo A, Pinilla M, García V, et al Development of a panel of well-characterized human immunodeficiency virus type 1 isolates from newly diagnosed patients including acute and recent infections. AIDS Res Hum Retroviruses. 2009 1;25(1):93–102. doi: 10.1089/aid.2008.0174 19113978 10.1089/aid.2008.0174

[49] de Felipe B, Pérez-Romero P, Abad-Fernández M, Fernandez-Cuenca F, Martinez-Fernandez FJ, Trastoy M, et al Prevalence and resistance mutations of non-B HIV-1 subtypes among immigrants in Southern Spain along the decade 2000–2010. Virol J. 2011;8:416 doi: 10.1186/1743-422X-8-416 21871090 10.1186/1743-422X-8-416 PMC3170306

[50] Alvarez M, García F, Martínez NM, García F, Bernal C, Vela CM, et al Introduction of HIV type 1 non-B subtypes into Eastern Andalusia through immigration. J Med Virol. 2003 5;70(1):10–3. doi: 10.1002/jmv.10368 12629637 10.1002/jmv.10368

[51] Instituto Nacional de Estadística (INE) [Internet]. Available from: www.ine.es

[52] Brenner BG, Roger M, Stephens D, Moisi D, Hardy I, Weinberg J, et al Transmission Clustering Drives the Onward Spread of the HIV Epidemic Among Men Who Have Sex With Men in Quebec. J Infect Dis. 2011 10 1;204(7):1115–9. doi: 10.1093/infdis/jir468 21881127 10.1093/infdis/jir468 PMC3164430

[53] Delgado E, Cuevas MT, Vega Y, Montero V, Sánchez M, Carrera C, et al. Identificación de un cluster de subtipo A que se transmite entre hombres que tienen relaciones sexuales con hombres en diversas comunidades autónomas de España. In Málaga: VI Congreso Nacional de GESIDA y 8.a Reunión Docente de la RIS (SEIMC); 2014. p. 12. Available from: http://www.gesida-seimc.org/contenidos/congresos/anteriores/2014/gesida2014-VIcongresocomunicaciones.pdf

[54] Patiño Galindo JA, Torres-Puente M, Gimeno C, Ortega E, Navarro D, Galindo MJ, et al Expansion of the CRF19_cpx Variant in Spain. J Clin Virol Off Publ Pan Am Soc Clin Virol. 2015 8;69:146–9. 10.1016/j.jcv.2015.06.094 26209397

