# Supplementary material for: High prevalence and diversity of HIV-1 non-B genetic forms due to immigration in southern Spain: A phylogeographic approach
Source: PLoS One. 2017 Oct 30;12(10):e0186928. doi: 10.1371/journal.pone.0186928 (PMC5662216; doi:10.1371/journal.pone.0186928)
Supplement: S1 Table — (DOCX) [file pone.0186928.s001.docx]

| Subtypes or CRFs | Reference Sequences  (Accession ID) |
| --- | --- |
| Subtype A1 | DQ676872 |
|  | AB253421 |
|  | AB253429 |
|  | DQ823357 |
| Subtype A2 | AF286238 |
|  | GU201516 |
|  | AF286237 |
|  | AF286240 |
| Subtype B | K03455 |
|  | AY423387 |
|  | AY173951 |
|  | AY331295 |
| Subtype C | U52953 |
|  | U46016 |
|  | AF067155 |
|  | AY772699 |
| Subtype D | K03454 |
|  | AY371157 |
|  | AY253311 |
|  | U88824 |
| Subtype F1 | AF077336 |
|  | AF005494 |
|  | AF075703 |
|  | AJ249238 |
| Subtype F2 | AY371158 |
|  | AJ249236 |
|  | AJ249237 |
|  | AF377956 |
| Subtype G | AF084936 |
|  | AF061641 |
|  | U88826 |
|  | AY612637 |
| Subtype H | AF190127 |
|  | AF190128 |
|  | AF005496 |
|  | FJ711703 |
| Subtype J | EF614151 |
|  | GU237072 |
|  | AF082394 |
|  | AF082395 |
| Subtype K | AJ249235 |
|  | AJ249239 |
| CRF01_AE | GQ477441 |
|  | GU564221 |
|  | U54771 |
|  | AB032740 |
| CRF02_AG | AY271690 |
|  | AB485636 |
|  | L39106 |
|  | AB231896 |
| CRF03_AB | AF193276 |
|  | AF193277 |
|  | AF414006 |
|  | AF413994 |
| CRF04_cpx | AF049337 |
|  | AF119820 |
|  | AF119819 |
|  | FJ388917 |
| CRF05_DF | AF076998 |
|  | AF193253 |
|  | AY227107 |
|  | AF247518 |
| CRF06_cpx | AF064699 |
|  | AY535659 |
|  | AB286851 |
|  | AB485660 |
| CRF07_BC | EF368372 |
|  | AF286230 |
|  | AF286226 |
|  | AX149647 |
| CRF08_BC | HM067748 |
|  | AY008715 |
|  | AB746342 |
|  | AB773885 |
| CRF09_cpx | AJ866553 |
|  | AY093605 |
|  | AY093603 |
|  | AY093607 |
| CRF10_CD | AF289548 |
|  | AF289549 |
|  | AF289550 |
|  | AM260231 |
| CRF11_cpx | AF492624 |
|  | AF492623 |
|  | AJ291718 |
|  | AF179368 |
| CRF12_BF | AF408629 |
|  | AF408630 |
|  | AF385936 |
|  | AF385935 |
| CRF13_cpx | DQ845388 |
|  | DQ845387 |
|  | AF460972 |
|  | AF460974 |
| CRF14_BG | AF450096 |
|  | AF450097 |
|  | GU230137 |
|  | AF423756 |
| CRF15_01B | DQ354120 |
|  | AF516184 |
|  | AF530576 |
|  | AF529572 |
| CRF16_A2D | AY945736 |
|  | AF286239 |
|  | KT022403 |
|  | KT022406 |
| CRF17_BF | EU581825 |
|  | EU581827 |
|  | AY037281 |
|  | EU581828 |
| CRF18_cpx | AF377959 |
|  | AY586541 |
|  | AY894993 |
|  | KP718931 |
| CRF19_cpx | AY588971 |
|  | AY588970 |
|  | AY894994 |
|  | KU685581 |
| CRF20_BG | AY586545 |
|  | AY586544 |
|  | DQ020274 |
|  | KT276270 |
| CRF21_A2D | AY945737 |
|  | AF457051 |
|  | AF457072 |
| CRF22_01A1 | AY371159 |
|  | GQ229529 |
|  | AY371165 |
|  | EU743963 |
| CRF23_BG | AY900571 |
|  | AY900572 |
| CRF24_BG | AY900574 |
|  | AY900575 |
|  | FJ670526 |
|  | KC473838 |
| CRF25_cpx | EU693240 |
|  | EU697906 |
|  | EU697908 |
|  | DQ826726 |
| CRF26_AU | FM877780 |
|  | FM877782 |
|  | FM877777 |
|  | FM877781 |
| CRF27_cpx | AJ404325 |
|  | AM851091 |
|  | AM851090 |
|  | AM041039 |
| CRF28_BF | DQ085872 |
|  | DQ085873 |
|  | DQ085874 |
|  | JF804809 |
| CRF29_BF | DQ085876 |
|  | AY771590 |
|  | DQ085871 |
|  | JF804806 |
| CRF31_BC | EF091932 |
|  | AY727526 |
|  | AY727527 |
| CRF32_06A1 | AY535660 |
|  | DQ167215 |
| CRF33_01B | AB547464 |
|  | DQ366659 |
|  | DQ366662 |
|  | AB547463 |
| CRF34_01B | EF165541 |
|  | EF165539 |
|  | EF165540 |
| CRF35_AD | EF158043 |
|  | EF158040 |
|  | EF158041 |
|  | EF158042 |
| CRF36_cpx | EF087995 |
|  | EF087994 |
|  | KR017774 |
| CRF37_cpx | EF116594 |
|  | AF377957 |
|  | JF683745 |
|  | KP718917 |
| CRF38_BF | FJ213781 |
|  | FJ213782 |
|  | FJ213780 |
|  | JN235962 |
| CRF39_BF | EU735534 |
|  | EU735536 |
|  | EU735535 |
| CRF40_BF | EU735538 |
|  | EU735540 |
|  | EU735539 |
|  | EU735537 |
| CRF42_BF | EU170155 |
|  | EU170136 |
|  | EU170138 |
|  | EU170139 |
|  | EU170141 |
| CRF43_02G | EU697904 |
|  | EU170136 |
|  | EU697907 |
|  | EU697909 |
| CRF44_BF | FJ358521 |
|  | AY536235 |
| CRF45_cpx | FN392874 |
|  | FN392875 |
|  | FN392876 |
|  | FN392877 |
| CRF46_BF | DQ358801 |
|  | DQ358802 |
|  | HM026456 |
|  | HM026457 |
| CRF47_BF | GQ372987 |
|  | FJ670529 |
|  | KC473839 |
|  | KC473840 |
| CRF49_cpx | HQ385477 |
|  | HQ385479 |
|  | HQ385478 |
