# Supplementary material for: High prevalence and diversity of HIV-1 non-B genetic forms due to immigration in southern Spain: A phylogeographic approach
Source: PLoS One. 2017 Oct 30;12(10):e0186928. doi: 10.1371/journal.pone.0186928 (PMC5662216; doi:10.1371/journal.pone.0186928)
Supplement: S2 Table — (DOCX) [file pone.0186928.s002.docx]

**Supplementary Table 2:** Evolutionary rates for each of the main HIV-1 lineages found in this study obtained through Bayesian phylogenetic inference.

| Subtypes or CRFs | Evolutionary rate (s/s/y) (x 10^-3^) (95% confidence interval) |
| --- | --- |
| Subtype A | 2.7 (1.8-3.6) |
| Subtype C | 1.9 (1.1-2.7) |
| Subtype F | 2.2 (1.1-3.3) |
| Subtype G | 3.1 (2-4.2) |
| CRF02_AG | 3.5 (2.5-4.5) |

CRF: Circulating Recombinant Form; s/s/y: substitutions/site/year
