# Supplementary material for: High prevalence and diversity of HIV-1 non-B genetic forms due to immigration in southern Spain: A phylogeographic approach
Source: PLoS One. 2017 Oct 30;12(10):e0186928. doi: 10.1371/journal.pone.0186928 (PMC5662216; doi:10.1371/journal.pone.0186928)
Supplement: S3 Table — (DOCX) [file pone.0186928.s003.docx]

| *Variables* | Subtype A1 | | | |  |
| --- | --- | --- | --- | --- | --- |
|  | **B (ET)** | **Wald** | **OR (95% CI)** | **p** |  |
| Gender (Male) | 1.82 (0.82) | 4.94 | 6.17 (1.24 - 30.73) | **0.026** |  |
| Age |  |  |  |  |  |
| < 35 | Ref. |  |  |  |  |
| 35-45 | 0.88 (0.83) | 1.13 | 2.42 (0.48 - 12.30) | 0.288 |  |
| >45 | 0.57 (0.97) | 0.34 | 1.77 (0.26 - 11.85) | 0.558 |  |
| Risk factor (HTX) | 19.96 (0.47) | 0.01 | 4.66 (0-6.98) | 0.998 |  |
| Nationality |  |  |  |  |  |
| Others | Ref. |  |  |  |  |
| Africa | -2.48 (0.93) | 7.04 | 0.08 (0.01 - 0.52) | **0.008** |  |
| Eastern Europe | 1.21 (0.94) | 1.65 | 3.35 (0.53 - 21.12) | 0.199 |  |
| Location Area |  |  |  |  |  |
| Others | Ref. |  |  |  |  |
| El Ejido | -0.81 (0.92) | 0.77 | 0.45 (0.07 - 2.70) | 0.379 |  |
| Granada | -0.20 (0.98) | 0.04 | 0.82 (0.12 - 5.54) | 0.836 |  |
| Viral Load | 0.00 (0.00) | 0.33 | 1.00 (1.00 - 1.00) | 0.565 |  |
| CD4 count | 0.01 (0.01) | 0.07 | 1.00 (0.99 - 1.00) | 0.796 |  |
| Sampling interval |  |  |  |  |  |
| 2005-2007 | Ref. |  |  |  |  |
| 2007-2009 | 1.37 (1.21) | 1.28 | 3.94 (0.37 - 42.26) | 0.257 |  |
| 2009-2011 | 3.08 (1.32) | 5.41 | 21.75 (1.62 - 291.39) | **0.02** |  |
| 2011-2013 | 2.20 (1.10) | 4.00 | 9.01 (1.05 - 77.71) | **0.045** |  |
| Constant | -23.65 (8.47) | 0.01 |  | 0.998 |  |
|  | Chi-square model = 60.8, df = 13, p < 0.001.  Nagelkerke *R*^2^ = 0.57 | | | |  |
