# Supplementary material for: High prevalence and diversity of HIV-1 non-B genetic forms due to immigration in southern Spain: A phylogeographic approach
Source: PLoS One. 2017 Oct 30;12(10):e0186928. doi: 10.1371/journal.pone.0186928 (PMC5662216; doi:10.1371/journal.pone.0186928)
Supplement: S4 Table — (DOCX) [file pone.0186928.s004.docx]

|  | Number | Median GD | Minimum GD | Maximum GD |
| --- | --- | --- | --- | --- |
| A1 | 51 | 0.022 | 0.000 | 0.041 |
| C | 30 | 0.046 | 0.008 | 0.067 |
| F1 | 30 | 0.036 | 0.000 | 0.074 |
| G/CRF14_BG | 28 | 0.031 | 0.001 | 0.053 |
| CRF02_AG | 112 | 0.011 | 0.000 | 0.034 |
| All subtypes | 970 | 0.093 | 0.000 | 0.187 |
